# Hyperglycemia, oxidative stress, and the diaphragm: a link between chronic co-morbidity and acute stress?

**DOI:** 10.1186/cc13913

**Published:** 2014-06-10

**Authors:** Sebastian Hafner, Peter Radermacher, Manfred Frick, Paul Dietl, Enrico Calzia

**Affiliations:** 1 Sektion Anästhesiologische Pathophysiologie und Verfahrensentwicklung, Klinik für Anästhesiologie, Universitätsklinikum, 89081 Ulm, Germany; 2 Institut für Allgemeine Physiologie, 89081 Ulm, Germany

## Abstract

It is well established that prolonged, controlled mechanical ventilation is associated with contractile dysfunction of the diaphragm due to impaired function of the mitochondrial respiratory chain as a result of aggravated oxidative and nitrosative stress. Sepsis and circulatory failure induce a similar response pattern. Callahan and Supinski now show that streptozotocin-induced insulin-dependent diabetes causes a comparable response pattern, both with respect to function and physiology - that is, reduced fiber force and, consequently, muscle contractility - but also as far as the underlying mechanisms are concerned. In other words, the authors elegantly demonstrate that the consequences of a chronic metabolic disease and that of acute critical illness may lead to the same phenotype response. It remains to be elucidated whether the underlying co-morbidity (for example, diabetes) adds to or even synergistically enhances the effect of an acute stress situation (for example, sepsis, mechanical ventilation). In addition, extending their previous work during shock states, the authors also show that administration of a preparation of the enzymatic anti-oxidant superoxide dismutase can reverse the deleterious effects of diabetes. These data are discussed in the context of the fundamental role of hyperglycemia in relation to metabolism-dependent formation of reactive oxygen species.

## 

In this issue of *Critical Care* Callahan and Supinski [1] report on the effects of insulin-dependent diabetes on diaphragmatic function and morphology. Two weeks after induction of insulin-dependent diabetes by injection of streptozotocin, a drug that relatively specifically damages pancreatic β cells due to uptake via the glucose transport protein GLUT2, the resulting significant hyperglycemia (430 versus 110 mg · dL^-1^ in the control group) led to a one-third decrease in diaphragm mass associated with a 40% fall in specific force resulting from a comparable reduction in single fiber force. On the cellular level, this effect of hyperglycemia coincided with a marked increase in oxidative and nitrosative stress as assessed by protein carbonyls and tyrosine nitration, respectively. Injection of polyethylene glycol (PEG) superoxide dismutase (SOD; 2,000 IU · kg^-1^ intraperitoneally daily from day 8 until the end of the 2-week observation period) did not correct hyperglycemia but restored muscle force in all fiber types tested. The latter finding agrees well with nearly 20-year-old data that the anti-oxidant *N*-acetylcysteine restored the otherwise dose-dependent streptozotocin-induced impairment of diaphragmatic force despite hyperglycemia being unchanged [2]: hyperglycemia-related excess production of reactive oxygen and nitrogen species rather than the high glucose level *per se* causes diaphragmatic dysfunction associated with insulin-dependent diabetes.

First of all, the reader may query the link between these effects of diabetes and critical illness. It is well established that prolonged mechanical ventilation induces contractile dysfunction of the diaphragm [3] due to impaired function of the mitochondrial respiratory chain as evidenced by reduced activity of both citrate synthase and cytochrome c oxidase associated with mitochondrial oxidative damage [4]. Partial preservation of spontaneous respiratory activity can at least partially attenuate the diaphragmatic contractile dysfunction, even at comparable airway pressures [5,6]. The impairment of diaphragmatic force is by no means exclusively due to mechanical ventilation: experimental sepsis induced by endotoxin challenge [7] or cecal ligation and puncture [8] caused a comparable response pattern and, according to the pathophysiological role of reactive oxygen species, treatment with anti-oxidants restored normal diaphragmatic contraction [4,7-9]. The study by Callahan and Supinski now demonstrates that the consequences of a chronic metabolic disease and those of acute critical illness result in the same phenotype response. Moreover, this is true not only with respect to function and physiology - in this case reduced fiber force and muscle contractility - but also as far as the underlying mechanisms are concerned - that is, impaired activity of the mitochondrial respiratory chain due to enhanced oxidative and nitrosative stress. It is obvious that this analogy raises a subsequent question: does an underlying co-morbidity (for example, diabetes) add to or even synergistically enhance the effect of an acute stress situation (for example, sepsis, mechanical ventilation)? Future experiments may answer this question. Clearly, the time course of the co-morbidity will assume major importance in this context: in rats, depending on the interval between streptozotocin injection and tissue sampling, diaphragmatic fatigue was increased, normalized, or even attenuated [10].

What can we conclude from the present study in terms of therapeutic consequences? Callahan and Supinski convincingly demonstrate that injection of PEG SOD allowed for a near-complete reversal of the diabetes-induced diaphragmatic contractile dysfunction. Thereby, the authors extend their previous findings on the salutary effects of PEG SOD on diaphragmatic dysfunction induced by endotoxin, myocardial ischemia-related heart failure, phrenic stimulation, or prolonged mechanical ventilation as well as on endotoxin-induced cardiac dysfunction. Given its apparent therapeutic potential during various pathological conditions, does PEG SOD have potential for becoming widely used during critical illness? Previously, the equivocal role of oxygen radicals in general has been elegantly highlighted in this journal [11], and this radical paradox assumes particular importance for SOD: its substrate, the superoxide radical, can either initiate or terminate oxidative stress [12]. Moreover, genetic SOD over-expression did not affect organ dysfunction during resuscitated murine septic shock despite enhanced anti-oxidative capacity as demonstrated by reduced oxidative DNA damage, most likely as a result of the lack of a parallel rise in tissue activity of the downstream anti-oxidant enzyme catalase [13]. Finally, factors other than aggravated oxidative stress may also contribute to diaphragmatic dysfunction: sedation with propofol during well-maintained spontaneous breathing produced the same impairment of diaphragmatic contractility as 24 hours of mechanical ventilation [14], and in endotoxin-challenged rats mechanical ventilation even partly restored rather than increased diaphragmatic force generation [15]. In both studies, the degree of diaphragmatic contractile dysfunction was not directly related to the level of oxidative stress.

Finally, do the results of Callahan and Supinski limit the importance of hyperglycemia as a key factor of organ failure? Clearly, as in their previous studies, the authors demonstrated the effectiveness of PEG SOD despite unchanged blood glucose levels. Nevertheless, it is well established that hyperglycemia is associated with oxidative stress [16]. Moreover, the mechanical ventilation-induced diaphragmatic contractile dysfunction was caused by mitochondria-derived oxidative stress, which resulted, at least in part, from a 'metabolic substrate overflow' associated with down-regulation of 5'-adenosine monophosphate-activated protein kinase (AMPK) [4]. Maintenance of normoglycemia attenuated mitochondrial dysfunction and tissue apoptosis during prolonged critical illness in rabbits [17], and in mice with resuscitated, polymicrobial septic shock, controlling hyperglycemia improved liver tissue mitochondrial respiratory activity and tissue apoptosis at least in part due to AMPK activation [18].

## Abbreviations

AMPK: 5'-adenosine monophosphate-activated protein kinase; PEG: Polyethylene glycol; SOD: Superoxide dismutase.

## Competing interests

The authors declare that they have no competing interests.
